# Amphiphilic Polyurethane with Cluster-Induced Emission for Multichannel Bioimaging in Living Cell Systems

**DOI:** 10.1021/acsmacrolett.3c00657

**Published:** 2023-12-26

**Authors:** Nan Jiang, Ke-Xin Li, Jia-Jun Wang, You-Liang Zhu, Chang-Yi Zhu, Yan-Hong Xu, Martin R. Bryce

**Affiliations:** † Key Laboratory of Preparation and Applications of Environmental Friendly Materials, Key Laboratory of Functional Materials Physics and Chemistry of the Ministry of Education, Jilin Normal University, Changchun 130103, China; ‡ State Key Laboratory of Supramolecular Structure and Materials, College of Chemistry, Jilin University, Changchun 130012, China; § Department of Chemistry, Durham University, Durham DH1 3LE, United Kingdom

## Abstract

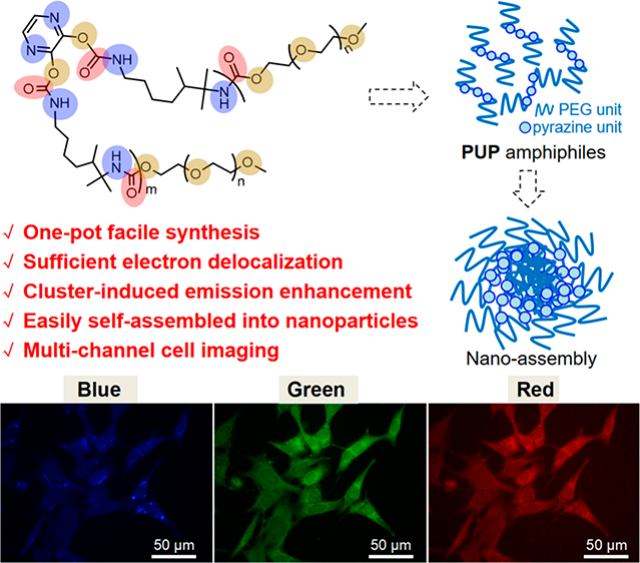

The development of single-component materials with low cytotoxicity and multichannel fluorescence imaging capability is a research hotspot. In the present work, highly electron-deficient pyrazine monomers were covalently connected into a polyurethane backbone using addition polymerization with terminal poly(ethylene glycol) monomethyl ether units containing a high density of electron pairs. Thereby, an amphiphilic polyurethane-pyrazine (**PUP**) derivative has been synthesized. The polymer displays cluster-induced emission through compact inter- and/or intramolecular noncovalent interactions and extensive through-space electron coupling and delocalization. Molecular rigidity facilitates red-shifted emission. Based on hydrophilic/hydrophobic interactions and excitation dependence emission at low concentrations, **PUP** has been self-assembled into fluorescent nanoparticles (**PUP** NPs) without additional surfactant. **PUP** NPs have been used for cellular multicolor imaging to provide a variety of switchable colors on demand. This work provides a simple molecular design for environmentally sustainable, luminescent materials with excellent photophysical properties, biocompatibility, low cytotoxicity, and color modulation.

The research and development of fluorescent materials have brought huge commercial and industrial benefits to today’s society. In recent years, the photoluminescence of nonconjugated luminescent polymers (NCLPs) in the aggregation state has been discovered, offering a new class of luminescent materials.^1−4^ Due to the advantages of simple synthesis, easy functionalization and low biotoxicity, NCLPs are showing prominent applications in photochemical sensing,^5,6^ information encryption,^7,8^ biomedicine,^9,10^ and so on. However, because NCLPs have flexible segment structures, their luminescence is generally limited to the short-wavelength region (blue or green light).^11−13^ There are few NCLPs with red-shifted wavelength emission.^14^ The mechanism for this remarkable photophysical phenomenon is still unclear and controversial, and substantially more data are needed to test the proposed models, which are based on restricted intramolecular motions. Obtaining NCLPs with red-shifted emission through reasonable structural design is currently a hot topic in the field of new luminescent materials and is of great significance for theoretical research and practical applications.

Excitation wavelength-dependent emission (EDE) of a luminescent material can be applied as a simple way to switch the emission color (wavelength). It has potential applications^15−18^ in the fields of photoelectric displays, biological probes and imaging, and dynamic anticounterfeiting. To date, a variety of EDE luminescent materials have been developed, such as single-molecule organic compounds,^19−21^ multicomponent composites,^22−24^ quantum dots,^25,26^ polymeric hydrogels,^27^ and metal complexes.^28−31^ However, these materials usually require harsh synthetic conditions (such as high concentration, high temperature, and high pressure), cumbersome operation conditions, high preparation cost, and poor stability. Moreover, the emission can be too weak at low concentrations and be restricted to a narrow adjustable range of wavelengths.^32−34^ Therefore, it is still a challenge to construct a single-component EDE material with a simple preparation, low cost, good biocompatibility, and excellent photophysical properties in low concentration applications.

Polyurethane (PU) is a typical NCLP containing repeated carbamate groups in the main chain, and PU has a wide range of industrial uses. The global production of PU in 2021 was >23 million tons, with the global market value of >63 billion US dollars, making PU a rational alternative to traditional materials.^35^ Moreover, because of its excellent biological and blood compatibility, biodegradability, and wear- and aging-resistant properties, PU and its derivatives are widely used in biomedical fields such as tissue-engineering stents, surgical dressings/pressure-sensitive adhesives, antibacterial surfaces, and catheters.^36−38^ At the same time, because of its good light stability, rich and diverse chemical composition and properties, PU derivatives have become popular new luminescent materials.^39−41^ Therefore, the development of luminescent polyurethane materials is of great significance for theoretical research into NCLPs and their practical application in biomedicine.

Based on the above background, pyrazine units with high electron deficiency and straight-chain poly(ethylene glycol) (PEG) monomethyl ether units with high-density shared electron pairs were introduced into a polyurethane main chain by a one-pot method, with the aim of promoting electronic communication and red-shifting the luminescence (Figure S1). This new polyurethanepyrazine (**PUP**) derivative has long-wavelength emission and self-assembly characteristics. The abundant electron-rich groups (carbonyl oxygen, alkyl oxygen, and amide units) in the polyurethane main chain can interact with the electron-deficient pyrazine chromophores, with sufficient electron coupling and delocalization to red shift the luminescence (Figure 1a). Generally, the water solubility of polymers and nanoparticles can be enhanced by coating them with surfactants, especially for biological applications.^42^ The addition of PEG surfactant units can improve the water solubility and reduce the biotoxicity of NPs by altering the molecules’ interactions with proteins.^43^ However, it should be noted that increasing the amount of PEG may reduce the cellular uptake of NPs and their efficacy as a drug delivery system. On the other hand, the limited stability of such noncovalent wrapped materials also has been a drawback.^43,44^ In the present work, PEG was attached covalently at the ends of the **PUP** chains to avoid the above problems. The resulting amphiphilic polyurethane can easily self-assemble into ultrastable **PUP** NPs through hydrophilic/hydrophobic interactions (Figure 1c) that readily disperse in water. The properties of **PUP** and its nanoparticles were characterized by gel permeation chromatography (GPC), UV–vis absorption spectroscopy, Fourier transform infrared spectroscopy (FT-IR), scanning electron microscopy (SEM), and dynamic light scattering (DLS). In addition, the cellular uptake and multichannel optical imaging of **PUP** NPs were investigated to evaluate their biological potential. **PUP** NPs were used for multicolor imaging of cells, benefiting from excellent biocompatibility and the EDE properties of aggregate luminescence.

**Figure 1:**
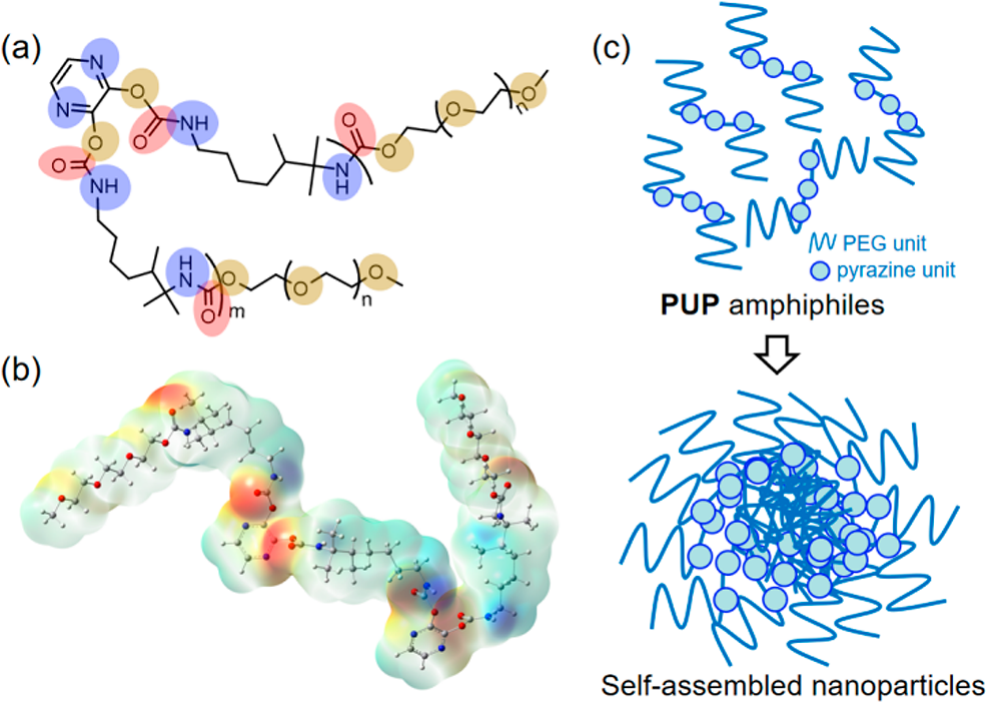
(a) Chemical structure of **PUP**. (b) Optimized molecular conformation and electrostatic potential map. (c) Proposed model for noncovalent bond assembly of **PUP** leading to formation of nanoparticles.

As shown in Figure S1, **PUP** was produced by a one-pot reaction with pyrazine-2,3-diol, trimethylhexamethylene diisocyanate, and poly(ethylene glycol) monomethyl ether. The chemical structure of **PUP** was characterized by ^1^H NMR and FTIR spectra (Figures S2 and S3). The results of powder X-ray diffraction (PXRD) also show that the unique steamed bun peaks of crystalline and amorphous states coexist (Figure S4). Figure 1b shows the molecular electrostatic potential diagram from which the delocalization of electrons in the nonconjugated **PUP** backbone can be intuitively understood. First, the electrostatic potential of the backbone is relatively continuous, which should be closely related to the multiple tight, noncovalent interactions present within the molecular chain. The oxygen atoms assume the role of electron donor, and the electrons delocalize to the −NH part of the main chain and near the electron-deficient pyrazine unit, respectively. The extended delocalization promotes the overlap of electron clouds, leading to rigid conformations due to restricted intramolecular motion and ultimately promoting luminescence.

After the polymerization reaction, the absorption of the PU derivative **PUP** significantly broadened, increasing the light utilization rate (Figure S5). Figure 2a shows the emission spectra of the **PUP** powder under different excitation conditions. With increased excitation wavelength, the emission peak sequentially redshifts from 429 to 516 nm, demonstrating EDE fluorescence, which is one of the characteristics of nonconjugated luminous polymers.^24,27^ In addition, the fine structure of the cluster luminescence peak of **PUP** indicates its rigid conformation. Rigid structures can inhibit nonradiative attenuation and further enhance luminescence intensity.^45^ More importantly, as shown in Figure S6, the emission spectrum of **PUP** still changes with the change of excitation wavelength at a concentration as low as 5 μg/mL. This is because **PUP** has an appropriate chain flexibility and an architecture which facilitates many kinds of noncovalent interactions within/between molecules. The formation of oxygen clusters and the extension of electron delocalization enhance the degree of compact aggregation of **PUP** chains, thereby promoting the formation of a variety of clustered luminescence centers, enabling **PUP** to retain a good EDE even at very low concentrations.

**Figure 2:**
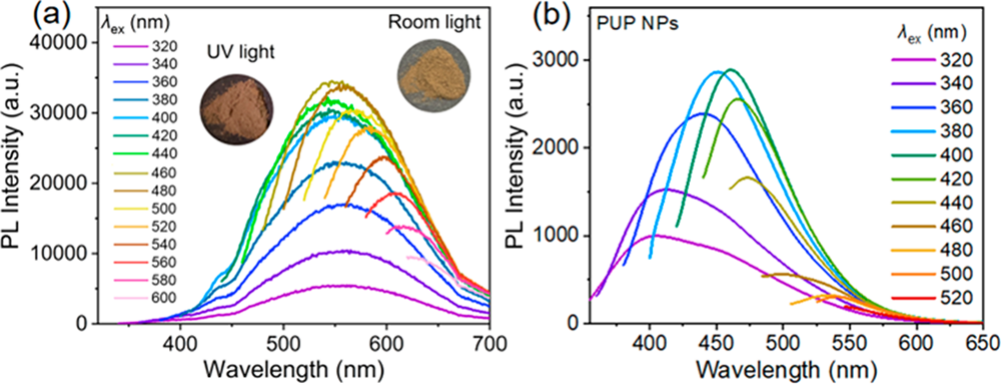
(a) Emission spectra of **PUP** powder with different excitation wavelengths at room temperature. Inset: Photos of **PUP** powder under room light and 356 nm UV light. (b) Emission spectra of **PUP** NPs (5 μg mL^–1^) in aqueous solution with different excitation wavelengths at room temperature.

To understand the influence of polymerization on the photophysical properties of the materials, the microscopic morphology was studied before and after polymerization. SEM images of the same amount of pyrazine monomer (20 mg/mL) and **PUP** after polymerization are shown in Figure 3. The monomer has a relatively dispersed blocky irregular microstructure (Figure 3a); however, **PUP** showed many relatively regular and tight flower-shaped nanowires/sheets (Figure 3b,c). Compared with large nanoblocks, close-knit nanostructures are more conducive to inter- and/or intrachain interactions and electron transport. Because the molecular chains of NCLPs are relatively soft, the aggregation behavior is variable and the aggregation forms and photophysical properties are tunable.

**Figure 3:**
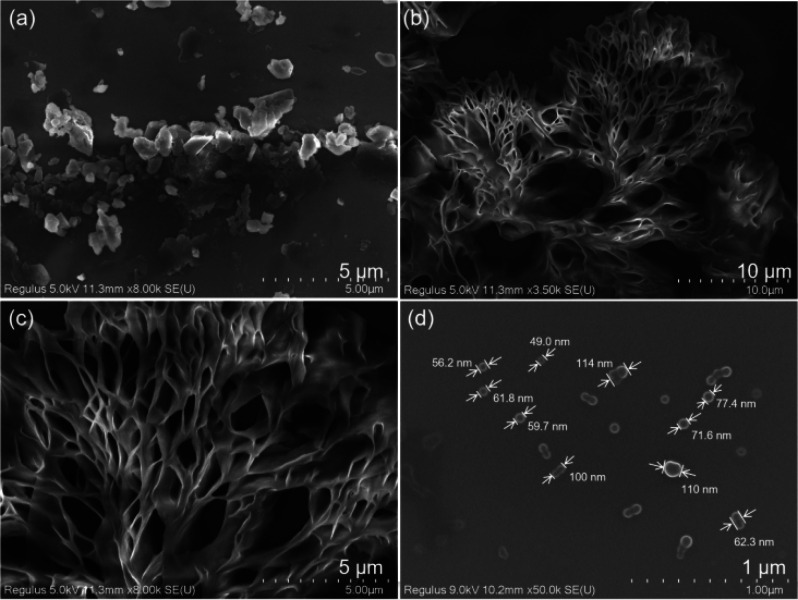
SEM images of 20 mg mL^–1^ (a) pyrazine-2,3-diol, (b, c) **PUP** dispersed in ethanol, and (d) **PUP** NPs in aqueous solution (5 μg mL^–1^).

To improve the water solubility of **PUP** for biological applications, nanoparticles were prepared by a solvent exchange method as described in the Supporting Information. The concentration of **PUP** NPs prepared is 5 μg/mL according to the standard curve (Figure S7). Figure 2b shows that the emission of **PUP** NPs maintains a good excitation dependence. Moreover, the emission intensity after self-assembly is higher than that of **PUP** in DMSO solution at the same concentration (Figures 2b and S6). The self-assembled **PUP** NPs had considerable quantum efficiency (0.6%) even at very dilute concentrations (5 μg/mL) in aqueous solution (Table S1). The above results suggest that after self-assembly, **PUP** has a more rigid structure and thus exhibits excellent light-emitting properties at a low concentration, which will be further discussed in the mechanism part later.

The size and morphology of NPs are critical for their biomedical applications. Therefore, the self-assembly behavior of **PUP** was examined by dynamic light scattering (DLS) and scanning electron microscopy (SEM). DLS (Figure S9) showed that the average hydrated kinetic particle size of the **PUP** NPs was 62 nm. The study of **PUP** NPs in aqueous solution on carbon-coated copper mesh by SEM showed spherical particle morphologies with a diameter of 49–110 nm and with an elliptical/heart-shaped nanosphere fusion (Figure 3d), which is consistent with the DLS results. **PUP** NPs of this size are well-suited to enter cells by endocytosis.^46^ In addition, spherical NPs are more likely to enter cells through endocytosis, compared with nonspherical NPs, thus facilitating a longer cycle time.^47^ After 15 days, SEM images showed that the micromorphology and size of the **PUP** NPs had barely changed (Figure S10), indicating that they have good stability. The high stability of the nanoparticles is conducive to their better circulation in the blood. Therefore, **PUP** NPs with good water solubility, suitable size and high stability can be applied in living cells.^47^ The introduction of poly(ethylene glycol) chains can reduce the cytotoxicity of organic materials. The negligible cytotoxicity of **PUP** NPs toward 4T1 cells (Figure S11) demonstrated the successful design of efficient and safe NPs.

Theoretical calculations for **PUP** were performed at the B3LYP/6-31G(d) level using Gaussian based on density functional theory (DFT). Figure S12 shows that the optimized molecular conformation of **PUP** has an appropriate structural distortion to favor multiple intra- and/or interchain interactions (e.g., C=O···H—C, N—H···N, and C—H···N hydrogen bonds, etc.) and short contacts, which are very conducive to aggregation and hence the restriction of intramolecular movement of the molecules in their excited states, ultimately promoting emission. Figure S13 shows the highest occupied and lowest unoccupied molecular orbital (HOMO and LUMO) diagrams of **PUP**. The HOMO and LUMO orbitals are located on both sides of the chain, respectively, showing spacially well-separated frontier molecular orbitals, indicating the possible existence of intramolecular charge transfer (ICT). To verify this, the emission spectra of **PUP** were obtained in different polarity solvents (Figure S8). The results showed that the emission red-shifted with increased solvent polarity, indicating ICT, which will bring spatial electron conjugation within the chains, conducive to luminescence.^14^ In addition, under the influence of hydrogen bonds, most of the oxygen atoms in **PUP** are less than 2.8 Å apart. It is suggested that oxygen atoms within and between molecular chains aggregate to form oxygen clusters^32^ (Table S2).

Molecular dynamics simulations further explored the sources of long wavelength emission and the excellent luminescence properties of **PUP** (Figure S14). The radial distribution function *g*(*r*) of C=N (N) and C=O (O) of **PUP** was obtained. As shown in Figure 4a, the distance between C=N (N) and C=O (O) atoms is in the range of 3.0 to 4.8 Å. The peak at 3.0 Å represents the interaction between C=N (N) and C=O (O) in the **PUP** chain; the peaks at 3.5 and 4.8 Å represent the interaction of C=N (N) and C=O (O) between different **PUP** chains. It is proved that N on the pyrazine unit plays an important role in both interchain and intrachain aggregation behavior. The radial distribution function *g*(*r*) of N and C=O (O), C—O—C (O), and O—C=O (O) of **PUP** was also obtained. Their first peaks correspond to 2.3, 2.4, and 3.0 Å, respectively, and the remaining peaks are shown in Figure S15, with the purple line representing the ether-oxygen chains, which interact weakly with N due to its greater distance. The interaction between N and oxygen in C=O is the strongest. The excitation dependent fluorescence will result from the combined functional groups, such as C=O, N—H, and C—O, of **PUP** chains that give rise to intra- and/or interchain *n*–π* and π–π* transitions with different energy levels within the various emissive clusters. Figure 4b shows clusters of different hierarchy in which various types of electronic transitions occur, and finally, energy level splitting and EDE are realized.

**Figure 4:**
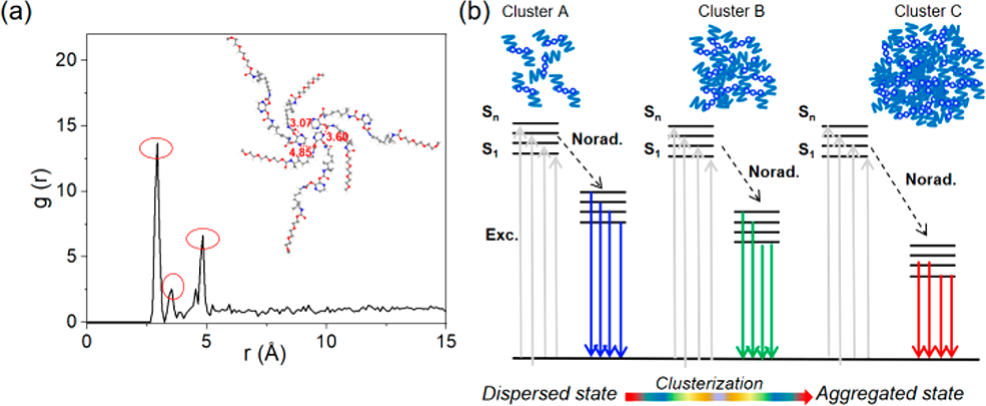
(a) Radial distribution function of C=N (N) and C=O (O) in **PUP** by molecular dynamics simulation. (b) The proposed mechanism of the EDE of **PUP**.

Inspired by the promising excitation-dependent fluorescence and good water solubility, we studied the multicolor cell imaging ability of **PUP** NPs. 4T1 cells were selected as a representative cell model for future development of anticancer drug delivery. 4T1 cells were incubated with 5 μg mL^–1^**PUP** NPs for 6 h and then observed under a confocal laser scanning microscope (CLSM). The images showed bright fluorescence in both the cell nucleus and cytoplasm of 4T1 cells under blue, green, and red channels (Figure 5a–c). The combined image (Figure 5d) shows light yellow fluorescence produced by the superposition of the three channels, clearly validating the material’s multicolor imaging capabilities.

**Figure 5:**
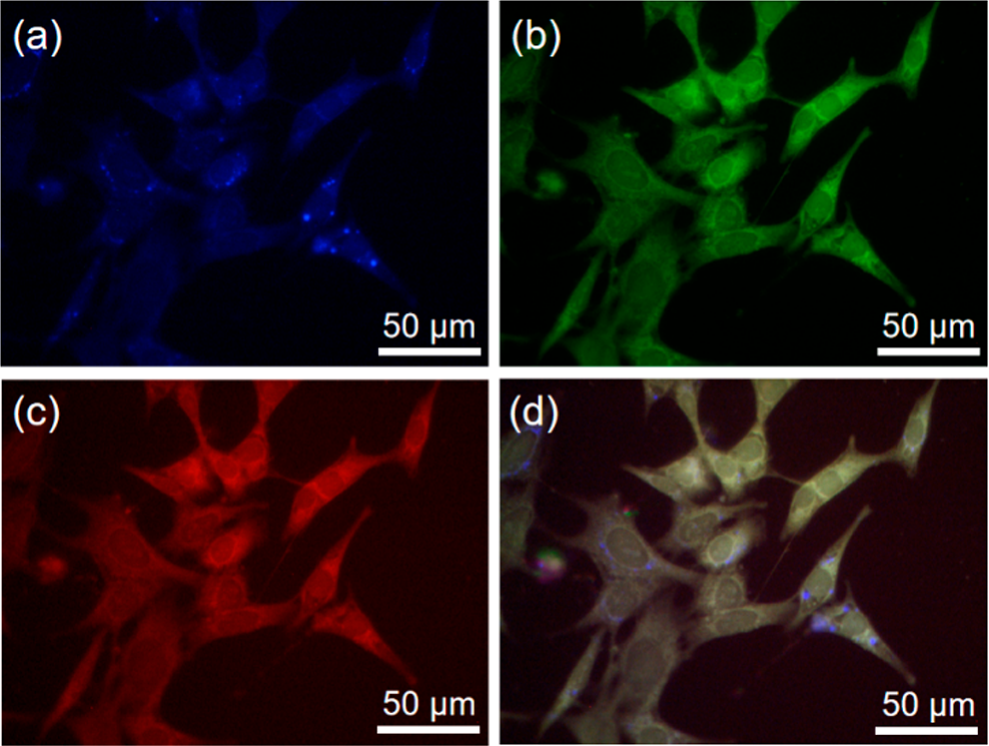
CLSM images of 4T1 cells incubated with **PUP** NPs (5 μg mL^–1^) for 6 h under (a) blue, (b) green, and (c) red channels in the cells. (d) The merged image of blue, green, and red channels.

In summary, in this work, a polymer-induced luminescence enhancement strategy has been used to covalently embed electron-deficient pyrazine units into a polyurethane main chain through a one-pot reaction. Then the PU chains were terminated by a polyethylene glycol monomethyl ether unit with a high density of electron pairs to give the target **PUP** material with electron donor and electron acceptor fragments. **PUP** displays excitation wavelength dependent colorful luminescence in the visible region. Water-soluble **PUP** NPs were constructed by a simple and mild self-assembly strategy without the need for additional surfactant. **PUP** NPs retained EDE in a dilute aqueous solution. **PUP** NPs were efficiently taken up by 4T1 cells, and color-tunable cell imaging with multiple channels has been demonstrated. This work should further inspire the exploitation of aggregation-induced luminescence based on restricted intramolecular motions of single-component polyurethane derivatives for biomedical and other emerging applications.^48^ The piperazine skeleton plays an important role in the development of new biological targets for the treatment of various diseases.^49^ However, there are fewer reports on pyrazine fragments in the field of medicinal chemistry.^50,51^ This study can promote the application of pyrazine derivatives in rational drug design and nanomedicinal chemistry research.

## Supporting Information Available

Additional structural characterization; Copies of NMR spectra; FT-IR spectra; Additional photophysical data; Scanning electron microscopy (SEM); Powder X-ray diffraction (PXRD) data; Computational data (PDF)
