# Supplementary material for: Amphiphilic Polyurethane with Cluster-Induced Emission for Multichannel Bioimaging in Living Cell Systems
Source: ACS Macro Lett. 2023 Dec 26;13(1):52–7. doi: 10.1021/acsmacrolett.3c00657 (PMC10795471; doi:10.1021/acsmacrolett.3c00657)
Supplement: Supplementary file 1 — mz3c00657_si_001.pdf [file mz3c00657_si_001.pdf]

Supporting Information

Amphiphilic Polyurethane with Cluster-Induced Emission for Multichannel Bioimaging in Living Cell Systems

Nan Jiang,[a] Ke-Xin Li,[a] Jia-Jun Wang,[a] You-Liang Zhu,[b] Chang-Yi Zhu,[a] Yan-Hong Xu[*a] and Martin R. Bryce[*c]

[a] Key Laboratory of Preparation and Applications of Environmental Friendly Materials, Key Laboratory of Functional Materials Physics and Chemistry of the Ministry of Education (Jilin Normal University), Changchun, 130103, China.
[b] State Key Laboratory of Supramolecular Structure and Materials, College of Chemistry, Jilin University, Changchun, 130012, China.
[c] Department of Chemistry, Durham University, Durham, DH1 3LE, UK
E-mails: xuyh198@163.com; m.r.bryce@durham.ac.uk

Contents:

1. Experimental details

2. Structural characterization

3. Photophysical properties

4. PUP nanoparticles studies

5. Theoretical calculations

6. References

1. Experimental details

General

The UV-vis absorption spectra were recorded on a Shimadzu UV-3100 spectrophotometer. The fluorescence spectra were recorded on a Hitachi F-4700 spectrometer and Edinburgh Instruments FLS-1000 spectrometer. The fluorescence lifetimes (τ) and fluorescence quantum yields were recorded using an Edinburgh Instruments FLS-1000 spectrometer. Using an integrating sphere to obtain all the light emitted by the sample, the quantum yield is determined by comparing the number of emitted photons with the number of absorbed photons. ^{1}H NMR spectra were recorded on a Varian 500 MHz spectrometer. The ^{1}H NMR spectra were referenced internally to the residual proton resonance in DMSO-d_6 (δ 2.5 ppm). The molecular weights of the polyurethane were determined by gel permeation chromatography (GPC) on a Waters 410 instrument with monodispersed polystyrene as the reference and THF as the eluent at 35 °C. Scanning electron microscope (SEM) images were obtained using a JEOL model JSM-6700 instrument operating at an accelerating voltage of 5.0/6.0/9.0 kV. The samples were prepared by placing microdrops of the solution on a holey carbon copper grid. Powder X-ray diffraction (PXRD) data were recorded on a Rigaku model RINT Ultima III diffractometer by depositing powder on a glass substrate.

All density functional theory (DFT) calculations were performed using the Gaussian 16 C.01 program at B3LYP/6-31G(d) level.[1]

Molecular dynamics simulation calculation method

In Materials Studio (MS) the initial model of the molecule was constructed using the "Amorphous Cell" module, and the initial density was set to 1.0 g/cm^3. Periodic boundary conditions were used, i.e., boxes were used in MS to represent the environment outside the molecule. 35 molecules were invested in the construction process, resulting in a total of 10 AC boxes. The structure was then subjected to 10,000 energy-minimization iterations using the Smart algorithm to rule out unreasonable contact situations, such as overlapping parts and overly dense contact between molecules. In this step, the conformation with the lowest energy was selected as the starting point for the subsequent molecular dynamics simulation. Next, NPT dynamics simulations were used to obtain the physical properties of the system, such as density,

volume, kinetic energy, and potential energy, resulting in the final equilibrium structure. NPT dynamics simulations were conducted at 298.15K for a total duration of 5 nanoseconds with 1 femtosecond per time step, resulting in 50001 models.

In the simulation, the Dreiding force field was used to calculate the interatomic interactions within the system. The long-range electrostatic interaction terms were solved by the Ewald summation method with an accuracy of 0.001 kcal·mol^{-1}. The van der Waals interaction force was calculated using an atom-based method with a cut-off distance of 12.5 Å. At the same time, to control the system temperature, the Nose-Hoover thermostat and the Berendsen constant pressure were used to maintain the pressure stability. All molecular dynamics simulations were performed using a time step of 1 femtosecond. In summary, in this research process, the initial model was built using Amorphous Cell module in Materials Studio. Through steps such as energy minimization and dynamic simulation, the balanced structure and physical properties of the system were obtained. The interaction was calculated using the Dreiding force field, with appropriate temperature and pressure control, providing strong support for further molecular modelling studies.[2,3]

Preparation method of water-soluble nanoparticles

The water-soluble nanoparticles were prepared by solvent exchange method. Firstly, PU derivative **PUP** (1 mg) was dissolved in methanol (1 mL), then the mixture was slowly dropped into deionized water (10 mL) and the methanol was volatilized by stirring at a constant speed for 12 h. After the methanol was completely volatilized, the mixture was put into the dialysis bag for dialysis, and the residual methanol was removed. Then, a 0.22 μm filter head was used to filter to further obtain uniformly dispersed nanoparticles. Then the concentration of nanoparticles was calculated using the standard curve.

Cell culture method

Mouse breast cancer cells (4T1 cells) were selected as the cell type for this experiment. First, Roswell Park Memorial Institute (RPMI) 1640 medium containing 10% fetal bovine serum by volume was configured, and the cell culture vial was placed in an incubator at a temperature of 37 ℃ and 5% CO_2 for culture. In order to ensure that the cells have sufficient nutrients, the medium was changed every two days.

Cell imaging

Confocal laser scanning microscopy (CLSM) was used for imaging of the material on the cells, and a 1 mL cell suspension was added to the confocal petri dish at a density of 50,000 cells per well. The cell culture vial was placed in the incubator overnight. The original medium was extracted, 1 mL of medium containing material (10 μg mL^{-1}) was added, and cultured in the incubator for 3 h. The cell imaging of the material was observed by CLSM.

Cytotoxicity test method

The cytotoxicity of the materials was determined by 3-(4,5-dimethylthiazol-2-yl)-2,5-diphenyl-2*H*-tetrazolium bromide (MTT) assay. 4T1 cells were placed into 96-well plates at a density of 10,000 cells per well, that is, 100 μL cell suspension was added into each well. The 96-well plates were incubated overnight in an incubator for cell adhesion growth. The media were then removed, and then media containing different concentrations of materials (0-40 μg mL^{-1}) were added to the cell pore plates, each 100 μL. The 40 μg mL^{-1} group was used as the blank control group, and the cells were cultured in the incubator for 24 h. Then 10 μL of MTT (5 mg mL^{-1}) was added to each well and cultured in an incubator for 4 h. The medium was replaced with DMSO (200 μL). The absorbance at a reference wavelength of 490 nm was recorded on an enzyme-labeler.

Synthesis of PUP

A mixture of pyrazine-2,3-diol (2.62 mmol, 0.294 g), poly(ethylene glycol) monomethyl ether (M_w = 200 g mol^{-1}; 1.98 mmol, 0.396 g), anhydrous THF (2 mL), anhydrous DMSO (4 mL), trimethylhexa-1,6-diyl diisocyanate (3.61 mmol, 0.826 g) and DABCO (0.105 mmol, 0.012 g) were added to a dried two-neck round-bottom flask. The solution was heated at 75°C for 18 h under nitrogen atmosphere. After that time the clear solution became significantly viscous, indicating the polymerization reaction had occurred. The crude product was dissolved in chloroform and then reverse precipitated from excess diethyl ether. Then the product was dried under vacuum for 24 h to obtain the resulting **PUP** (0.475 g); yield 41%. ^{1}H NMR (500 MHz, DMSO-d_6, δ [ppm]): 6.25 (s, 2H), 4.03 (s, 4H), 3.4-3.6 (broad, PEG protons), 3.23 (s, 3H; PEG terminal -OCH_3 protons), 2.6-3.1 (broad, 2H), 0.7-1.6 (broad, 15H). FTIR: 3431 cm^{-1}(N-H),

2890 and 2966 cm^{-1}(-CH_2- asymmetric and symmetric stretch), 1743 (C=O), 1047 cm^{-1} (C-O-C stretch PEG). M_p: 9367; M_n: 12686; M_v: 15252; M_w: 15863; PD: 1.2504.

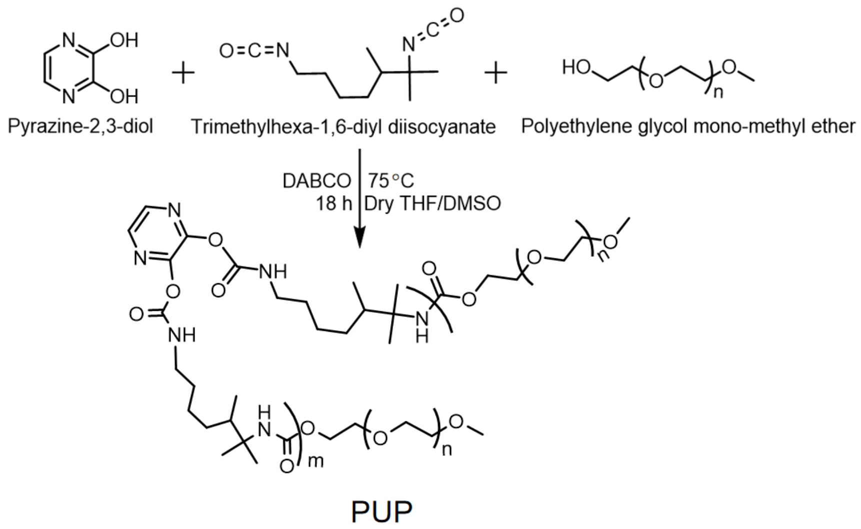

Figure S1. Synthetic route to the polyurethane derivative **PUP**.

2. Structural characterization

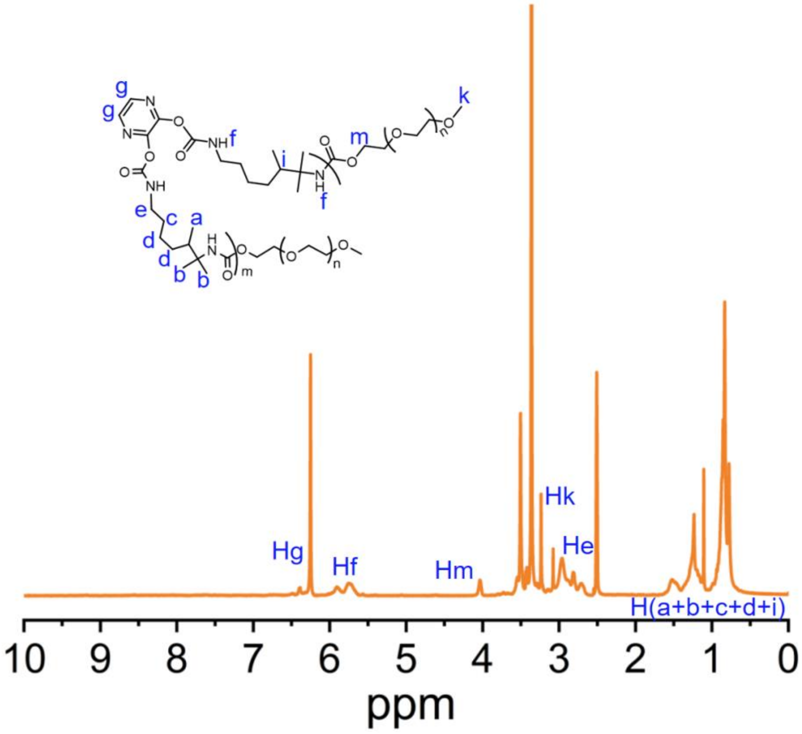

Figure S2. ^{1}H NMR spectrum of **PUP** in DMSO-d_6 with residual proton resonance in DMSO-d_6 (δ 2.5 ppm).

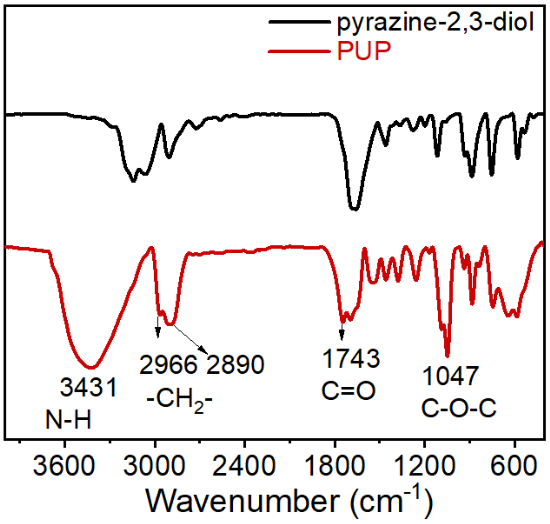

Figure S3. FTIR spectra of pyrazine-2,3-diol and **PUP** powder.

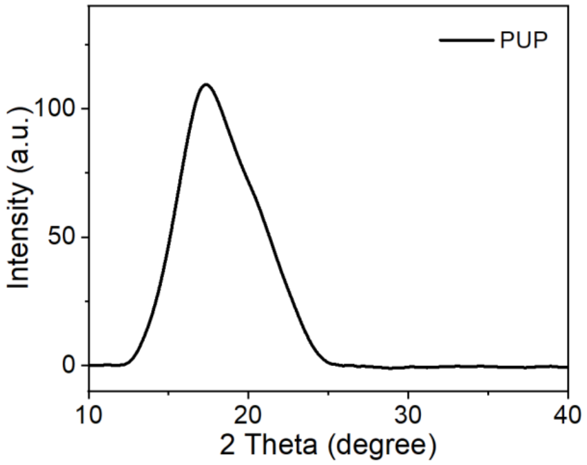

Figure S4. XRD pattern of **PUP** powder.

3. Photophysical properties

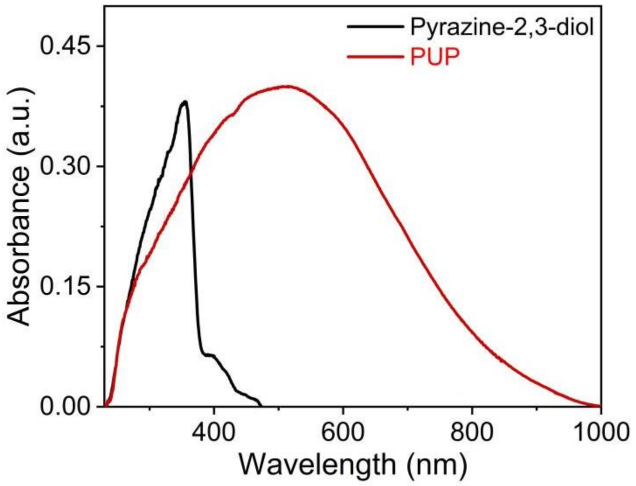

Figure S5. Absorption spectra of pyrazine-2,3-diol and **PUP** powders.

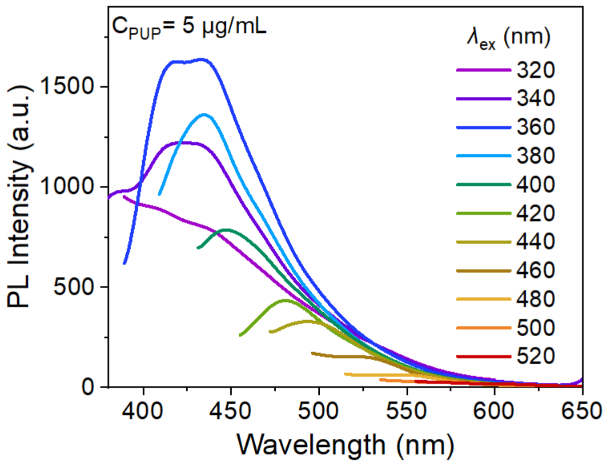

Figure S6. PL spectra of 5 μg/mL **PUP**/DMSO solution at varying λ_{ex}.

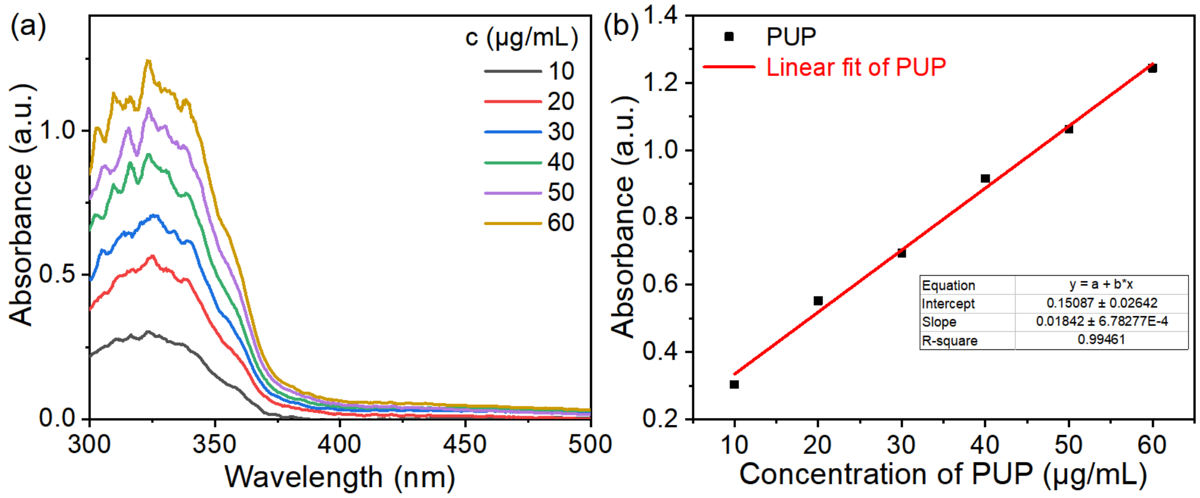

Figure S7. (a) Absorption spectrum of **PUP** NPs aqueous solution at different concentrations. (b) Standard curve of **PUP** NPs aqueous solution.

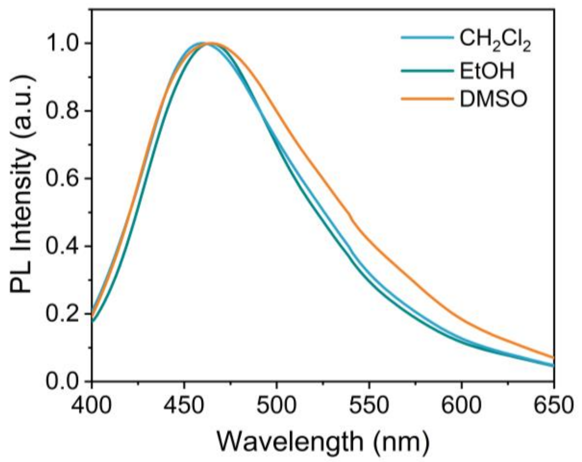

Figure S8. Emission spectra of **PUP** in dichloromethane (CH_2Cl_2), ethanol (EtOH) and dimethyl sulfoxide (DMSO) solvent.

4. PUP nanoparticles studies

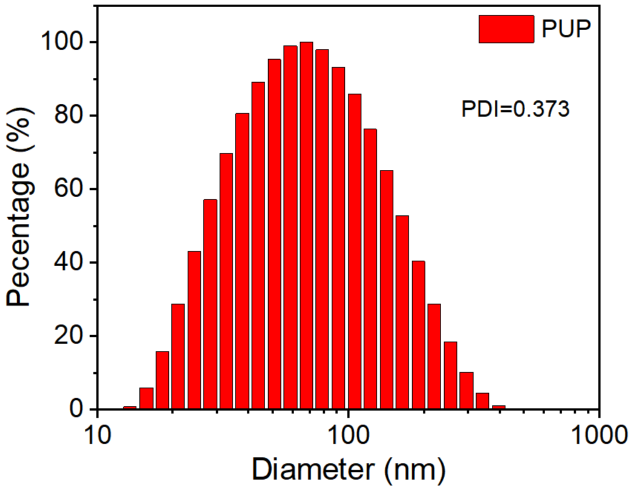

Figure S9. DLS particle size map of **PUP** NPs aqueous solution at room temperature.

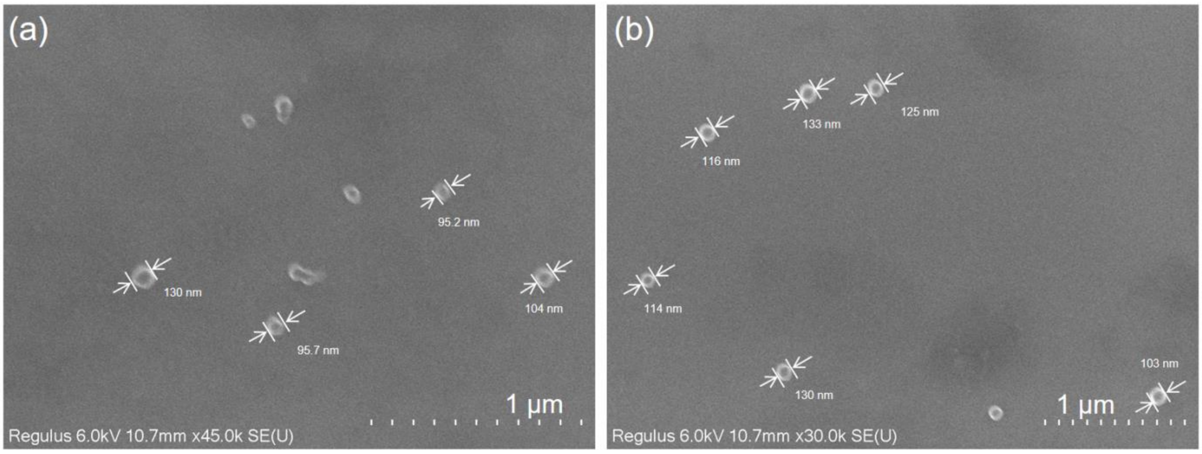

Figure S10. Both (a) and (b) are SEM images of 5 μg mL^{-1} **PUP** NPs aqueous solution after 15 days of storage.

Table S1. The fluorescence lifetime (LT) and quantum efficiency (QY) of **PUP** powder, 5 μg mL^{-1} **PUP** solution in DMSO and 5 μg mL^{-1} **PUP** NPs aqueous solution (λ_{ex}=365 nm).

	LT	QY
Powder	2.70 ns (λ_{em}=480 nm)	0.8%
Solution	1.17 ns (λ_{em}=423 nm)	0.1%
NPs	1.73 ns (λ_{em}=445 nm)	0.6%

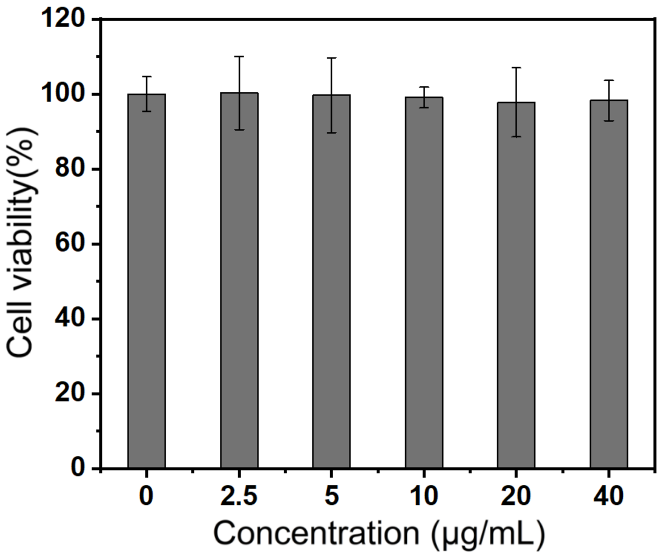

Figure S11. Relative viability of 4T1 cells after 24 h co-incubation with different concentrations of **PUP** NPs aqueous solutions.

5. Theoretical calculations

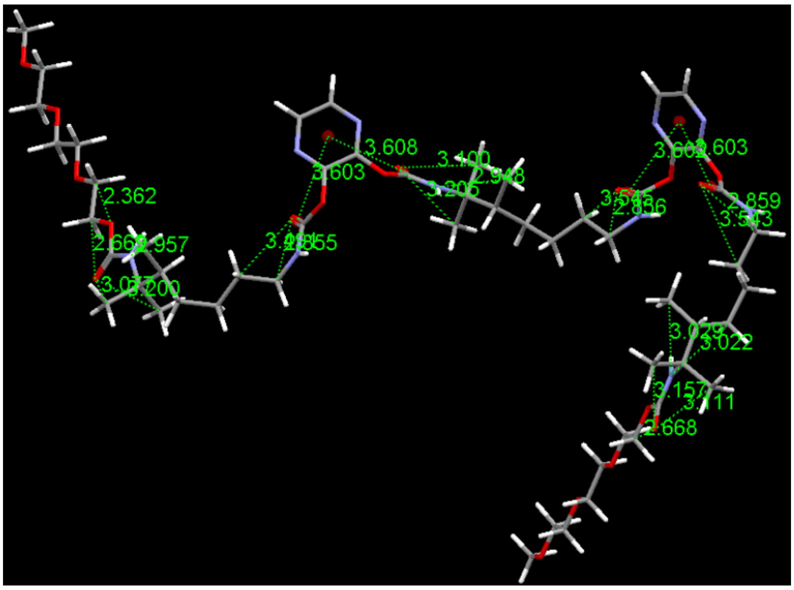

Figure S12. Theoretical calculations based on **PUP** at the B3LYP/6-31G(d) level.

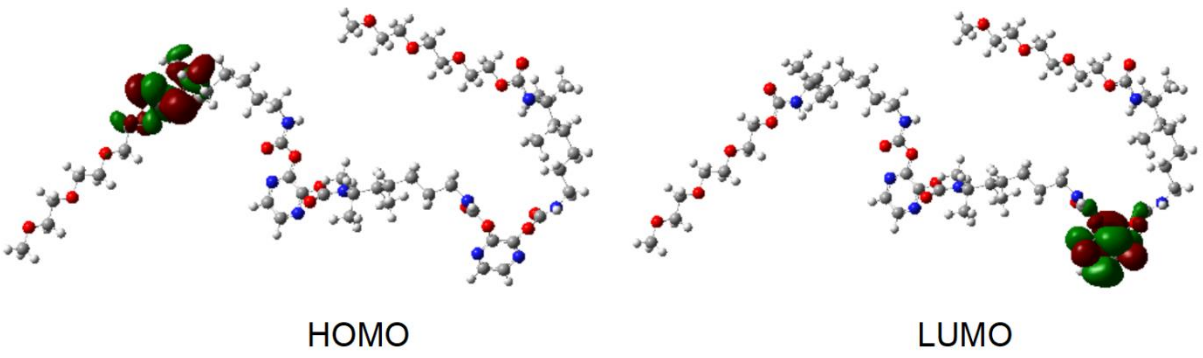

Figure S13. HOMO-LUMO orbital diagram of **PUP**.

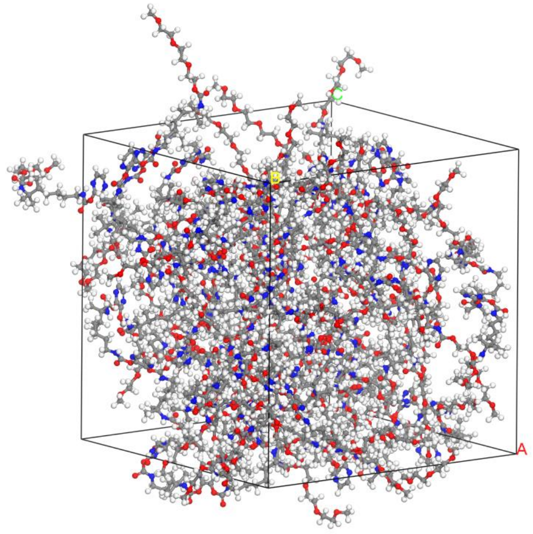

Figure S14. Snapshot of the simulation box of the **PUP** at 5 ns last frame by molecular dynamics simulation.

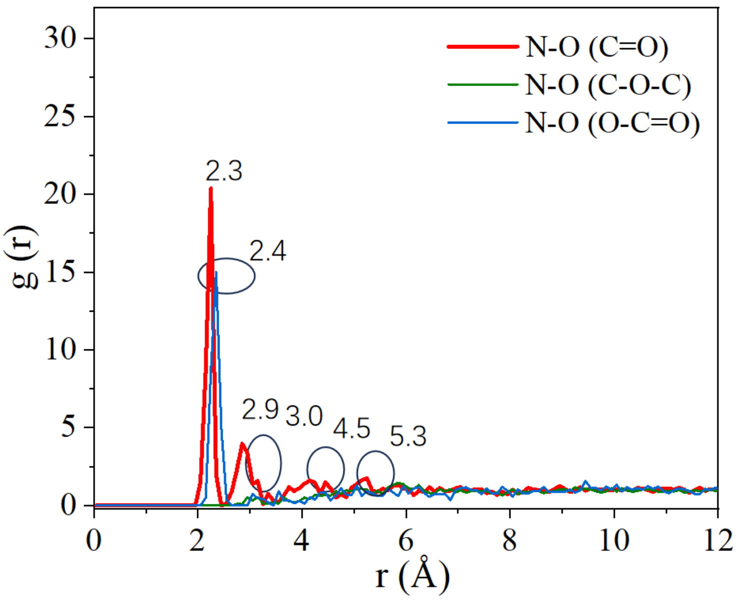

Figure S15. Radial distribution function of N and C=O (O), C-O-C (O), O-C=O (O) in **PUP** by molecular dynamics simulation.

Table S2. Conformational parameters of optimized model of **PUP**.

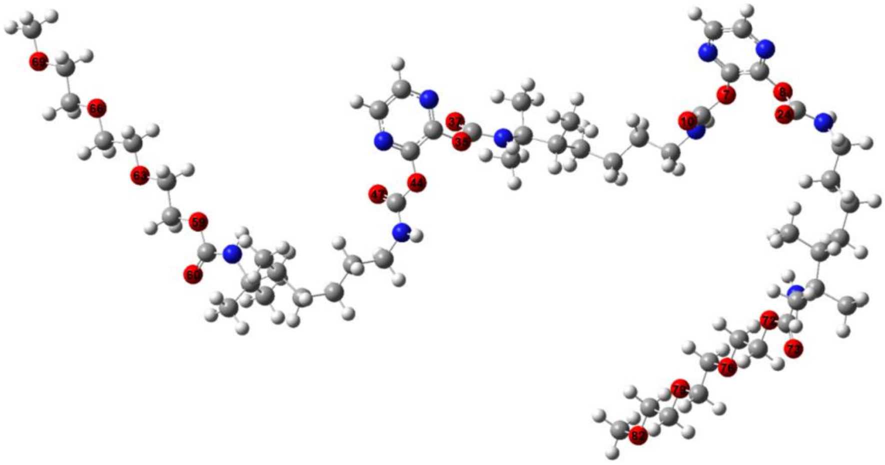

	Distance (Å)
O7-O8	2.74
O10-O7	2.29
O24-O8	2.29
O59-O63	3.59
O47-O44	2.29
O44-O36	2.74
O36-O37	2.29
O66-O69	3.59
O63-O66	3.59
O59-O60	2.27
O72-O73	2.27
O72-O76	3.59
O76-O79	3.59
O79-O82	3.59
O36-O44	2.74

6. References

1. Frisch, M. J.; Trucks, G. W.; Schlegel, H. B.; Scuseria, G. E.; Robb, M. A.; Cheeseman, J. R.; Scalmani, G.; Barone, V.; Petersson, G. A.; Nakatsuji, H.; Li, X.; Caricato, M.; Marenich, A. V.; Bloino, J.; Janesko, B. G.; Gomperts, R.; Mennucci, B.; Hratchian, H. P.; Ortiz, J. V.; Izmaylov, A. F.; Sonnenberg, J. L.; Williams-Young, D.; Ding, F.; Lipparini, F.; Egidi, F.; Goings, J.; Peng, B.; Petrone, A.; Henderson, T.; Ranasinghe, D.; Zakrzewski, V. G.; Gao, J.; Rega, N.; Zheng, G.; Liang, W.; Hada, M.; Ehara, M.; Toyota, K.; Fukuda, R.; Hasegawa, J.; Ishida, M.; Nakajima, T.; Honda, Y.; Kitao, O.; Nakai, H.; Vreven, T.; Throssell, K.; Montgomery, J. A., Jr.; Peralta, J. E.; Ogliaro, F.; Bearpark, M. J.; Heyd, J. J.; Brothers, E. N.;

Kudin, K. N.; Staroverov, V. N.; Keith, T. A.; Kobayashi, R.; Normand, J.; Raghavachari, K.; Rendell, A. P.; Burant, J. C.; Iyengar, S. S.; Tomasi, J.; Cossi, M.; Millam, J. M.; Klene, M.; Adamo, C.; Cammi, R.; Ochterski, J. W.; Martin, R. L.; Morokuma, K.; Farkas, O.; Foresman, J. B.; Fox, D. J. Gaussian 16, Revision C.01, Gaussian, Inc., Wallingford CT, 2016.

2. Brehm, M.; Kirchner, B. *J. Chem. Inf. Model.* **2011**, *51*, 2007–2023.

3. Gurina, D.; Surov, O.; Voronova, M.; Zakharov, A. *Nanomaterials* **2020**, *10*, 1256.
